# Late Type of Bronchial Response to Milk Ingestion Challenge: A Comparison of Open and Double-Blind Challenge

**DOI:** 10.1155/2012/515267

**Published:** 2011-11-03

**Authors:** Zdenek Pelikan

**Affiliations:** Allergy Research Foundation, Effenseweg 42, 4838 BB Breda, The Netherlands

## Abstract

*Background*. In some asthmatics the food allergy, for example, to milk, can participate in their bronchial complaints. The role of food allergy should be confirmed definitively by food ingestion challenge performed by an open challenge with natural foods (OFICH) or by a double-blind placebo-controlled food challenge (DBPCFC). *Objectives*. To investigate the diagnostic value of these techniques for confirmation of a suspected milk allergy in bronchial asthma patients. *Methods*. In 54 asthmatics with a positive history and/or positive skin tests for milk the 54 OFICH, and DBPCFC, were performed in combination with spirometry. *Results*. The 54 patients developed 39 positive late asthmatic responses (LAR) and 15 negative asthmatic responses to OFICH and 40 positive LARs and 14 negative responses to DBPCFC. The overall correlation between the OFICH and DBPCFC was statistically significant (*P* < 0.01). *Conclusions*. This study has confirmed the existence of LAR to milk ingestion performed by OFICH and DBPCFC in combination with spirometry. The results obtained by both the techniques did not differ significantly. The OFICH with natural food combined with monitoring of objective parameter(s), such as spirometry, seems to be a suitable method for detection of the food allergy in asthmatics. The DBPCFC can be performed as an additional check, if necessary.

## 1. Introduction

Food allergy is a clinical manifestation of an immunologic process in which foods or their components acting as antigen(s) stimulate the production of specific antibodies or sensitize the particular T lymphocyte subsets and then interact with them [1–11]. This interaction then induces a number of intracellular and extracellular processes, defined as a hypersensitivity mechanism(s), resulting in the manifestation of the clinical symptoms [1–23]. 

Principally, various types of hypersensitivity can be involved in food allergy; however, the immediate type (IgE-mediated) hypersensitivity has mostly been investigated and documented [1–9, 11–19, 24–28]. Nevertheless, in recent years evidence has been found for possible involvement of other hypersensitivity types, such as late type (Type III) and delayed type (Type IV) in the food allergy [1–3, 5–8, 14, 15, 23, 24, 29–39]. The exact immunopathologic mechanisms underlying various clinical manifestations of food allergy are, however, not yet fully clarified [1–3, 7–9, 15]. 

Food allergy can occur in two basic forms: a primary form, where the foods act as the primary and sole cause of the activation of the immunologic mechanism(s), and a secondary form, where food participates in an already existing hypersensitivity mechanism(s) activated by different antigens, for example, inhalant antigens. The secondary form occurs more frequently [1, 6–9, 11, 18, 40, 41]. Although the provocation tests with foods are not always performed routinely, they may be considered to be the definite confirmation of involvement of particular foods in the patient's complaints [1–13, 18, 20, 22, 25, 27, 28, 40, 42–51].

Provocation tests with foods can be performed using three basic techniques, the open food ingestion challenge (OFICH), double-blind placebo-controlled food challenge (DBPCFC), and the single-blind food ingestion challenge (SBFIC), all of them having a number of advantages and disadvantages [1–13, 18–22, 25–28, 40, 42–58].

The DBPCFC is generally considered to be “a golden standard” [1–13, 16, 25–28, 48, 49, 51, 58]. However, under some circumstances and for some reasons the OFICH may be more preferable to DBPCFC [1, 2, 6–11, 20–22, 25, 40, 43–46, 52, 53]. The purpose of this study was (a) to verify the possible involvement of milk allergy in some patients with bronchial asthma, (b) to compare the results attained by both the techniques, OFICH and DBPCFC, and to assess their suitability and diagnostic values for the confirmation of food allergy involvement in patients with bronchial asthma, by monitoring the objective parameters, such as lung function using spirometry.

## 2. Material and Methods

### 2.1. Patients

Fifty-four patients suffering from perennial bronchial asthma suspected of participation of milk allergy, examined at our Department of Allergology & Immunology, Institute of Medical Sciences “De Klokkenberg,” Breda, The Netherlands, and developing 39 positive late or 15 negative asthmatic responses to OFICH, volunteered to participate in this study.

These patients, 18–39 years of age, included 46 subjects suffering from already existing bronchial asthma due to various inhalant allergens in whom the milk has been suspected to participate possibly in their bronchial complaints and/or demonstrating positive skin tests with milk and 8 subjects in whom the milk has been suspected to be a sole cause of their bronchial complaints. They showed positive skin (prick and/or intradermal) tests with milk to various degrees, and in some of them also positive specific IgE antibodies for some foods have been recorded (Tables 1 and 2). They did not suffer from any airway infections and did not use oral corticosteroids or immunotherapy.

The patients were examined by routine diagnostic procedure, acting also as an exclusion-inclusion check, consisting of (1) general part: disease history, physical examination, basic laboratory tests, X-ray of the chest and sinuses, lung function, blood gases determination, bacteriological examination of the sputum; (2) allergologic part: skin tests with inhalant and food allergens, bronchial histamine thresholds [59], blood leukocyte differential count, determination of the serum immunoglobulins; (3) 95 bronchial provocation tests (BPT) with inhalant allergens [60–63]; (4) 54 OFICH with milk suspected from history and/or positive skin tests. The 54 food challenges with milk were then repeated by means of DBPCFC. A 5-day interval was always inserted between the consecutive tests to prevent the carryover effects and to allow the patient's recovery. 

All challenges were performed in a period without manifest symptoms and during a short hospitalization of the patients. The milk and all dairy products were avoided by the patients for 3-4 weeks before the challenges.

Inhaled glucocorticosteroids (*n* = 35), long-acting *β*_2_-sympathomimetics (*n* = 19), and oral cromolyn (*n* = 2) were withdrawn 4 weeks, inhaled cromolyn (*n* = 5), nedocromil sodium (*n* = 11), and leukotriene modifiers (*n* = 1) 2 weeks, and other treatments 48 hours before each of the challenges. The local ethical committee approved this study, and informed consent was obtained from all study participants.

### 2.2. Allergens

Dialyzed and lyophilized extracts of inhalant allergen as well as foods (Allergopharma, Reinbek, Germany) diluted in PBS were used for skin tests in concentrations 50–500 BU/mL, as indicated in the subsection “Skin tests.” The recommended concentrations by the manufacturer were 100–500 BU/mL for skin prick as well as for intracutaneous tests.

### 2.3. Skin Tests

The skin prick tests (SPTs) in concentrations of 500 BU/mL were performed [27, 28, 59, 64, 65], and evaluated after 20 minutes and 24 hours. If the SPTs were negative, then intracutaneous (intradermal) tests in concentrations of 100 BU/mL and 500 BU/Ml were carried out [1, 6–10, 18–22, 28, 57, 60–65] and evaluated 20 minutes, 6, 12, 24, 36, 48, 72, and 96 hours after the injection. If the SPT were positive (immediate/early skin response), then the intracutaneous tests were performed in concentrations of 50 BU/mL and 200 BU/mL and evaluated 20 minutes, 6, 12, 24, 36,48, 72 and 96 hours after the injection. Histamine diphosphate was used as a positive control, whereas PBS was used as a negative control. A skin wheal (>7.0 mm in diameter) appearing 20 minutes after the injection was considered to be positive immediate skin response, the skin infiltration observed between 6 and 12 hours was considered to be a late skin response, and the skin induration recorded later than 48 hours was designated a delayed skin response [6–10, 60–65].

### 2.4. Spirometry

The asthmatic responses were monitored by using spirometry (Spirograph D-75; Lode NV, Groningen, The Netherlands), recording the FVC and FEV_1_, and evaluated by the following criteria: (1) the decrease in FEV_1_ of less than 10% with respect to the prechallenge values as negative, from 10% to 20% as doubtful, and of 20% or more as positive asthmatic response; (2) the decrease in FEV_1_ values recorded at least at 3 consecutive time intervals was considered to be a positive response; (3) the response appearing within 2 hours after the challenge was considered to be an immediate asthmatic response (IAR), that occurring between 4 and 24 hours to be a late/asthma response (LAR), and response appearing later than 24 hours after the challenge to be a delayed asthmatic response (DYAR) [9, 18, 22, 60–63].

### 2.5. Food Used for the Ingestion Challenge

The quantities of milk used both for the OFICH and DBPCFC were similar to those consumed usually by the patients in order to obtain the highest degree of reproducibility. The amount of 100 mL of natural milk (3.5 g of protein and 3.5 g of fat per 100 mL) was used for the OFICH. The amount of 20 g of powdered whole milk (containing 3.0 g of protein and 2.9 g of fat) dissolved in 80 mL water was used for DBPCFC. The 5% glucose solution was used as control (placebo) for OFICH. For the DBPCFC, 20 g of tablet inactive ingredients, so-called “excipients” (including lactose, dibasic calcium, sucrose, maize corn, starch, and microcrystalline cellulose) dissolved in 80 mL water, was used as control (placebo). Both the solutions used for DBPCFC, the powdered milk as well as the inactive tablet mass (excipients) were enriched with 4 g of glucose to mask their taste. The control challenges were performed according to the same schedule as those with the experimental foods. The DBPCFC arrangement was in principle triple-blinded, and that both for the technician preparing the test material, and for the nurse performing the challenge, and lastly for the patient himself.

### 2.6. Schedule of the Food Challenge

The OFICH and DBPCFC challenges as well as the spirometry monitoring of were performed according to the European and international standard procedures [2, 4, 25–27, 40, 42, 48, 49, 57, 58] modified by us [6–10, 18–22], by the following schedule: (1) recording of the initial (baseline) values at 0, 5 and 10 minutes; (2) ingestion of the food within 10 minutes, followed by a 1-hour waiting interval to allow the food to be ingested. During this interval the parameters were measured four times to exclude an unexpected or too early reaction; (3) recording of the postchallenge values at 0, 5, 10, 20, 30, 45, 60, 90, and 120 minutes, and every hour up to the 12th hour and every second hour during the 22nd and 38th hour, the 46th and 58th hour intervals [6–10, 18–22].

### 2.7. Control Group

Twelve patients suffering from perennial bronchial asthma, developing 12 late asthmatic responses (LAR) to BPT with *Dermatophagoides pteronyssinus*, however demonstrating negative history, skin test, and RAST for the foods, volunteered to participate as controls. In 6 patients the OFICH and in 6 patients the DBPCFC were performed with the most frequently consumed food, usually milk, cheese or peanuts, according to the same schedule as applied in the patients studied.

### 2.8. Statistical Analysis

Asthmatic responses were analyzed by generalized multivariate analysis of the variance (MANOVA) model [66]. The polynomials were fitted to the mean curves over time (8 time points within 120 minutes and 14 time points up to 24 hours after the challenge), and the appropriate hypotheses were tested by the modified MANOVA computerized system.

In every patient the postchallenge FEV_1_ values measured at each time interval were compared with the prechallenge values and evaluated by Wilcoxon matched-pair signed-rank test. The mean postchallenge FEV_1_ values were compared with corresponding post-challenge control values at each of the time points and analyzed by the Mann-Whitney *U* test. The correlation between the OFICH and DBPCC was evaluated by Wilcoxon matched-pair signed rank test. A *P* value < 0.05 was considered to be statistically significant.

## 3. Results

**Figure 1:**
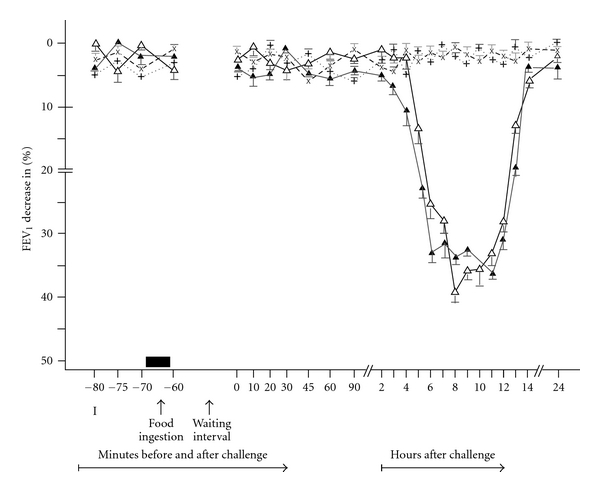
Late asthmatic responses due to the food ingestion challenge (LAR) in 39 patients with bronchial asthma. The mean percentage changes in the FEV_1_ calculated from all patients with positive LAR. LAR (*n* = 39) recorded after OFICH: experimental food (∆) and control food (x) and after DBPCFC: experimental food (▲) and placebo (+) I: initial (baseline) values; food ingestion: OFICH or DBPCFC; waiting interval: 1 hour; bars: means ± SEM.

**Figure 2:**
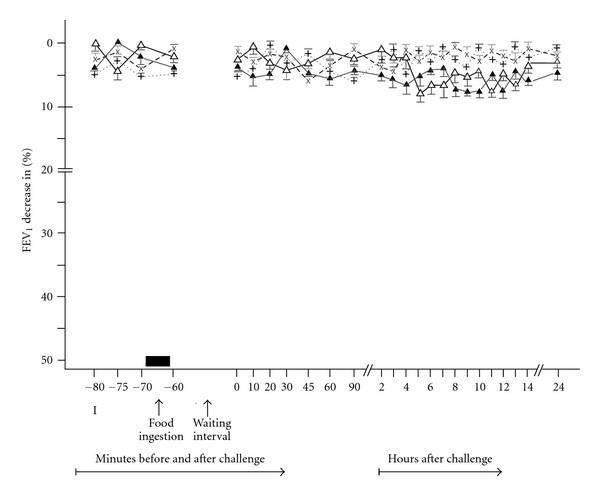
Negative asthmatic responses due to the food ingestion challenge (NAR) in 15 patients with bronchial asthma. The mean percentage changes in the FEV_1_ calculated from all patients with NAR. NAR (*n* = 39) recorded after OFICH: experimental food (∆) and control food (x) and after DBPCFC: experimental food (▲) and placebo (+) I: initial (baseline) values; food ingestion: OFICH or DBPCFC; waiting interval: 1 hour; bars: means ± SEM.

In 54 patients suffering from bronchial asthma, 54 open ingestion challenges with milk (OFICH) have resulted in 39 positive asthmatic responses of late type (LAR; *P* < 0.01) and 15 negative asthmatic responses (NAR; *P* > 0.1) (Table 1, Figures 1 and 2). The LARs began 4–6 hours, reached their maximum 6–10 hours, and resolved within 24 hours after the milk ingestion challenge (OFICH). The LARs were associated with various general and bronchial complaints, predominantly dyspnea (100%), wheezing (79%), cough (28%), oral itching (23%) and gastrointestinal complaints (31%) (Table 2), whereas no general or bronchial complaints were recorded during the NARs. The LARs as well as the NARs correlated with disease history, skin tests and other diagnostic parameters to various degrees (Tables 1, 2, and 3). The 39 patients developing positive LAR for milk in OFICH demonstrated positive skin tests, and positive (suspect) history in 48%, positive skin tests but unknown history in 4%, and positive (suspect) history but negative skin tests in 20%. The 15 patients developing negative asthmatic response (NAR) for milk in OFICH displayed positive skin test and suspect history in 11%, positive skin test but unknown history in 6%, and suspect history but negative skin tests in 11% (Tables 3 and 4). Survey of detailed agreement between the positive and negative asthmatic responses to OFICH with milk and the other diagnostic parameters (disease history, skin tests) in both the groups of patients, those with bronchial asthma to inhalant allergens and suspicion of milk allergy as well as those with bronchial asthma suspected of milk allergy only, is presented in Table 5. All 54 control ingestion challenges with glucose solution were negative (*P* > 0.1) and without any accompanying bronchial or general complaints.

The 54 patients challenged with milk by means of DBPCFC developed 40 positive LAR (*P* < 0.01) and 14 NAR (*P* > 0.05) (Table 6, Figures 1 and 2). The 38 of the 40 DBPCFC positive LARs correlated with the OFICH positive LARs (=97%; *P* < 0.01), whereas 13 of the 14 DBPCFC negative responses (NARs) correlated with the OFICH negative responses (NARs) (=87%; *P* < 0.05). 

The 3 noncorrelating cases showed 2 OFICH negative responses but DBPCFC positive LARs and 1 OFICH positive LAR but DBPCFC negative response. The overall correlation between the OFICH and DBPCFC responses was statistically significant (*P* < 0.01). All 54 DBPCFC control challenges with “tablet excipients” were negative (*P* > 0.1). No bronchial complaints were registered during the DBPCFC controls; however 1 patient developed diarrhea to a slight degree during this test (=2%).

### 3.1. Control Group

The 12 patients of the control group, in whom 6 OFICHs and 6 DBPCFCs with milk were performed, did not develop any asthmatic response. No general or bronchial complaints have been registered during these 12 NAR (*P* > 0.2).

## 4. Discussion

The role of foods and food allergy in bronchial asthma in producing bronchial complaints, especially bronchospasm, through the hypersensitivity mechanisms has already been investigated from various points of views [1–9, 11–13, 15, 16, 18, 20, 22, 26, 42, 45, 53, 55, 56, 67]. The involvement of food allergy in bronchial asthma, classically attributed to the IgE-mediated hypersensitivity upon involvement of IgE antibodies, mast cells, basophils, eosinophils, and Th_2_ lymphocytes, has mostly been investigated [1–3, 5, 9, 11–13, 15, 16, 24, 26, 29, 39, 44, 67]. Later, some evidence was also gathered for possible involvement of the non-IgE-mediated mechanism(s) upon participation of various cytokines, neutrophils, and Th_1_ lymphocytes in the food allergy events [5–9, 14, 15, 23, 24, 29–39, 43, 53, 54, 68, 69]. The link between the BALT and GALT and the disturbed homing of T and B lymphocytes (plasma cells) may also play an important role in these processes [1, 14, 15, 24, 29, 33, 39, 68, 69]. 

Patients with bronchial asthma upon participation of food allergy, having been challenged with foods, may develop various types of asthmatic (bronchus-obstructive) response. The immediate/early (IAR), late (LAR), and delayed (DYAR) asthmatic responses to food ingestion challenge, described in our previous papers [9, 18, 20, 22] and some of these types reported also by other investigators [1–3, 11–13, 16, 20, 43–46, 53, 55, 56, 70], are in principle analogical to the three types of asthmatic response to the bronchial challenge with inhalant allergens [60–63]. The IAR, LAR and DYAR due to the food ingestion challenge differ substantially not only with respect to the possibly underlying immunologic mechanisms, but also in their clinical features, time course and association with other diagnostic parameters [1, 2, 7–9, 12, 13, 15, 18, 20, 22–24, 30–33, 38, 39, 53–56, 70]. 

The immediate/early asthmatic response (IAR/EAR) to foods, due to the immediate (IgE-mediated) hypersensitivity mechanism, has been investigated most frequently [1–5, 9, 11–13, 16, 24, 26, 39, 40]. The late asthmatic response to foods (LAR) has also been reported in the literature [53, 55, 56, 70]. However, the immunologic mechanism(s) underlying the LAR, especially the possible involvement of IgE-mediated or non-IgE-mediated hypersensitivity, is not yet sufficiently clarified [2, 9, 15, 23, 24, 29, 39]. In our previous studies we also have observed and described the DYAR response to food ingestion challenge, analogical to the DYAR to bronchial challenge with inhalant allergens [63, 71], in which the involvement of cell-mediated hypersensitivity mechanism (Type IV allergy) could be presumed [9, 15, 18, 20, 22, 63]. This presumption may be supported by other investigators' findings of possible role of various cell types, such as Th_1_-cells, neutrophils, macrophages, dendritic cells, and a number of cytokines, chemokines, and other factors, in the clinical manifestations of food allergy, especially in its role in bronchial asthma [2, 14, 15, 24, 29–31, 33–38, 53, 54, 56]. However, there is still a dearth of information concerning the clinical features of the various types of bronchial response resulting from the ingestion (challenge) as well as their association and correlation with other *in vivo *and *in vitro *diagnostic parameters in large groups of well-defined patients [1, 2, 6–9, 11–13, 40, 41, 43, 45, 47, 49, 67].

Diagnostic confirmation of the involvement of food allergy in the clinical manifestations, especially in the bronchial asthma, is not always an easy assignment. The diagnostic parameters being customarily used in the practice, such as disease history, skin tests, determination of the total and specific IgE antibodies in the serum (PRIST, RAST, ImmunoCAP), demonstrate various degrees of correlation with the clinical manifestations due possibly to food allergy, such as bronchial asthma. None of these parameters demonstrated sufficient and statistically significant diagnostic value to predict and/or to conform arbitrarily the role of foods and food allergy in bronchial asthma in a given patient [1–13, 18, 27, 28, 41–43, 45, 58, 67, 72]. 

The role of foods in the bronchial asthma can definitely be confirmed only by the ingestion challenge with the suspected food(s), during which the particular asthmatic response types can be recorded quantitatively in its dynamic course [1, 2, 7–9, 12, 18, 22, 25, 27, 28, 42–44, 46, 48–50, 53]. The food challenge is therefore a more reliable test for the detection of a bronchial reaction to foods and its clinical consequences than data obtained from single skin tests and/or RAST/ImmunoCAP tests [1, 2, 9, 12, 18, 22, 26, 39, 42, 44–48, 50, 57, 58, 72].

The importance and significance of the food challenge for the diagnostic confirmation of the food allergy has repeatedly been demonstrated in the literature [1–13, 16–22, 25–28, 30, 39, 40, 42–46, 48–50, 52–55, 57, 58, 70, 72]. Unfortunately, papers dealing with the role of food allergy and food ingestion challenge in patients with bronchial asthma are not numerous [2, 9, 11–13, 16, 18–22, 26, 27, 42, 50, 52, 57, 58].

Nevertheless, the food ingestion challenge has also some limitations and contraindications and requires therefore some special conditions, and precautions [1, 2, 6, 9, 11, 13, 22, 24, 28, 40, 42–45, 47–50, 52, 57, 58]. The absolute contraindication is the suspected anaphylactic shock to the particular food(s), pregnancy, and any life-threatening disorder or situation, whereas the relative contraindication may be considered any state or disorder leading to any undesirable complication(s) or which can distinctly influence the food ingestion challenge results, such as treatment with certain drugs [1, 2, 6, 9, 22, 27, 28, 40, 42, 45, 48, 49, 52, 57, 58]. Food challenges, where the vital organ functions should be recorded, for example, lung function, severe diarrhea, or where a late onset of the response is expected, should be performed during hospitalization under standard conditions, and for a sufficiently long period of time, 56 hours at least [1, 6, 9, 22, 25, 27, 40, 48, 52, 53]. The foods, their parts and the related foods used for the ingestion challenge should be excluded from the diet for a sufficiently long period of time before the challenge, at least 7–14 days [1, 2, 6, 9, 18–22, 25, 27, 28, 40, 42, 45, 46, 48, 49, 52, 58].

The food ingestion challenge can be performed by an open challenge (OFICH) with natural foods, by double-blind placebo-controlled challenge (DBPCFC), or rarely by single-blind challenge (SBFIC), each of them having its advantages and disadvantages [1–13, 16, 18–22, 24–28, 39, 40, 42–52, 57, 58, 67, 70]. 

The OFICH with natural foods and suitable placebo is relatively simple easy to perform, the results are directly available, and no special processing of the foods used for the challenge is required. In addition, it is eligible in cases where the objective parameters can be recorded. This technique is not suitable for challenges where the results can be influenced by the patient or by the investigator or where the response can only be measured by means of subjective parameters, such as headache, (skin) itching, tiredness, and behavior changes [1–3, 6–11, 16, 22, 27, 42, 45, 48, 49, 52, 73]. 

The DBPCFC, considered by many investigators to be a “golden standard,” has a number of advantages as well as disadvantages [1–9, 25, 27, 42, 43, 48, 49, 51, 52, 57]. Its important advantage is exclusion of the influencing of results both by the patient and investigator, its usability in cases where the objective parameters cannot be recorded and the response can only be evaluated by means of subjective parameters, such as pain, headache, itching, gastrointestinal complaints, general malaise, behavior changes, and finally when the effects of drugs should be investigated [1–9, 25, 27, 28, 40, 42, 43, 46, 48, 49, 51, 52, 58]. 

Nevertheless, this technique has also some disadvantages, such as (a) processing of the food in a manner excluding its identification, which means the food must be colorless, tasteless, odorless. Such a preparation of foods can lead to essential changes of their structure and physical and/or chemical properties and sometimes it is not even possible; (b) providing a suitable placebo that matches the offending food in quantity and other properties is sometimes a technical problem; (c) the content of the capsules to be swallowed is maximally 500 mg; If the foods tested were administered in an amount equal to that used in a daily practice then the capsule number would increase enormously, otherwise the food will be taken in an amount less than the natural consumption; (d) the food administered in capsules excludes the oral cavity, tongue and oesophagus, organs which are often the site of the first reaction to foods; (e) by administering of food in capsules, the digestive process already beginning in the mouth is shifted to the gastric and duodenal mucosa and therefore prolonged; (f) the hidden placebo can sometimes induce a false-positive response [1–9, 25, 27, 42, 43, 48, 49, 51, 52, 57, 58]. 

The results of this study confirmed the existence of late type of asthmatic response (LAR) due to the food ingestion, which has already been described in our previous studies [7–9, 18, 20, 22] and reported by other authors [53, 55, 56, 70]. Although a possible role of an “IgE-mediated” hypersensitivity in the LAR caused by food allergy has already been suggested, the precise mechanism underlying this asthmatic response type is not yet sufficiently clarified [1–3, 5, 9, 13, 17, 18, 24, 27, 29, 34–39, 49, 53–55, 70]. These results have emphasized the importance of the food ingestion challenge for the diagnosis of food allergy in patients with bronchial asthma. The definite confirmation of this role should be provided by a food ingestion challenge combined with monitoring of lung function, for example, spirometry, demonstrating the particular types of asthmatic response in their dynamic course. 

Regarding the results of this study, together with our previous papers [6–10, 18–22] and other investigators' findings [2, 25, 28, 42–47, 49, 51, 53, 54, 57, 58] the diagnostic value of the food ingestion challenge seems to be superior to other diagnostic parameters. The significant correlation of the OFICH and DBPCFC results, both for the positive and for the negative asthmatic responses would suggest that in bronchial asthma, where the asthmatic responses can be measured by means of objective lung function, the DBPCFC is not superior to the OFICH [6–10, 20–22, 44–46, 52]. 

It can therefore be concluded that the OFICH combined with monitoring of objective diagnostic parameters, such as lung functions, can be considered to be definite confirmation of the suspected role of food allergy and involvement of certain food(s), such as cow's milk, in bronchial complaints of patients suffering from bronchial asthma. These patients may include both those suffering from bronchial asthma due to the inhalant allergens, in whom the food allergy is suspected as an additional cause of their bronchial complaints, and those in whom the food allergy, for example, for cow's milk, is suspected as an only cause of the bronchial asthma symptoms. The OFICH is a suitable and reliable technique in all cases of food allergy where the response can be measured by using the objective parameters and recorded for a sufficiently long period of time, such as 24–48 hours. In such cases this technique would be preferable, because it is easier, cheaper, quicker, and less burdening for the patient who will not need to swallow a large number of capsules.

The DBPCFC should be reserved for such cases, in which objective parameters cannot be measured; the response to food can only be expressed by subjective complaints, for example, itching, headache, tiredness, distinct discrepancy among the other diagnostic parameters that occur, or in cases in which the OFICH results are dubious or not reliable. Vice versa, in the cases in which the DBPCFC results seem to be unreliable, the OFICH can be performed as an extra check.

## Figures and Tables

**Table 1:** Characteristics of the patients and control subjects.

	Patients			Control subjects
	Total	LAR	NAR	
	*n* = 54	*n* = 39	*n* = 15	*n* = 12
Age (years)	31 ± 6	30 ± 7	32 ± 5	27 ± 6
Gender (M/F)	25/29	11/15	14/14	5/7
Disease history (years)	4.7 ± 1.3	3.5 ± 1.6	5.1 ± 1.0	4.2 ± 1.5
Asthmatic attacks per month	4 ± 1	5 ± 2	2 ± 1	3 ± 2
FEV_1_ (% predicted)	94.8 ± 4.2	93.1 ± 5.5	97.0 ± 3.3	95.6 ± 4.3
FVC (% predicted)	99.2 ± 1.1	96.4 ± 3.0	100.1 ± 1.4	98.0 ± 4.3
Blood leukocyte count (×10^9^/L)^●^	7.1 ± 0.8	7.5.0 ± 1.2	8.0 ± 0.4	7.9 ± 1.5
Blood eosinophil count (×10^6^/L)^●●^	355 ± 60	387 ± 56	329 ± 70	410 ± 53
Blood neutrophil count (×10^9^/L)^●●●^	5.2 ± 0.5	6.0 ± 0.3	4.5 ± 0.9	6.5 ± 0.4
Bronchial histamine threshold (BHT)^□^				
≤2.0 mg/mL		4	2	1
4.0 mg/mL		9	2	4
8.0 mg/mL		11	3	3
16.0 mg/mL		4	5	2
32.0 mg/mL		5	2	2
>32.0 mg/mL		6	1	0

Values: mean ± SD; ^●^: normal value = 4.0 − 10.0 × 10^9^/L; ^●●^: normal value ≤ 300 × 10^6^/L; ^●●●^: normal value: 2.0−7.2 × 10^9^/L;^ □^: normal value ≥ 32.0 mg/mL.

**Table 2:** Survey of other diagnostic parameters.

	Patients			Control subjects^▲^
	Total	LAR	NAR	
	*n* = 54	*n* = 39	*n* = 15	*n* = 12
Bronchial complaints ^∆^				
(i) Dyspnea		++	0	0
(ii) Wheezing		++	0	0
(iii) Cough		±	0	0
(iv) Expectoration		0	0	0
Positive skin response (SPT)^*◊*^				
(i) Immediate	31	24	7	1°
Negative skin response (SPT)^*◊*^	23	15	8	11
Positive skin response (i.c.)^*◊◊*^	37	28	9	0
(i) Immediate	−15	−12	−3	
(ii) Late	−21	−15	−6	
(iii) Delayed	−1	−1	−0	
Negative skin response (i.c.)	17	11	6	
Increased total IgE (serum)^□□^	1	1	0	0
Positive specific IgE (serum)^□□□^	9	7	2	1
Increased total IgG (serum)^●^	2	2	0	0
Increased sub-classes (serum)^●●^				
(i) IgG_1_	1	1	0	0
(ii) IgG_2_	0	0	0	0
(iii) IgG_3_	0	0	0	0
(iv) IgG_4_	2	1	1	0
Increased total IgM (serum)^●●●^	0	0	0	0
Increased total IgA (serum)^■^	3	3	0	0
Concomitant (allergic) disease				
(i) Allergic rhinitis	8	4	4	1
(ii) Atopic eczema	11	9	2	0
(iii) Urticaria	1	0	1	0
(iv) Angio-neurotic edema	0	0	0	1
(v) Gastrointestinal complaints	5	3	2	0

L: late asthmatic response; N: negative asthmatic response; ^ ∆^: Bronchial complaints accompanying the asthmatic response (author's modified score system): 0: absent, ±: very slight/incidental, +: slight, ++: moderate/intermittent, +++: pronounced/regularly, ++++: very pronounced/distinct/frequent; ^*◊*^: skin prick test (SPT) with milk extract; ^*◊◊*^: intracutaneous (intradermal) skin test with milk extract; ^□□^: total IgE in the serum (PRIST)-normal value ≤ 500 IU/mL; ^□□□^: positive allergen-specific IgE in the serum for milk (ImmunoCAP) ≥ 0.70 U/mL (=more than class 1); ^ ●^: total IgGin the serum (Single radial immunodiffusion and ELISA)-normal value ≤ 15.0 g/L; ^●●^: normal values: IgG_1_ < 5.0 g/L,IgG_2_ < 2.6 g/L, IgG_3_ < 0.4 g/L, IgG_4_ < 0.5 g/L; ^ ●●●^: IgM ≤ 3.8 g/L (<1.5); ^■^: IgA ≤ 4.0 g/L (<3.2); ^▲^: control subjects: 6 open (OFICH) + 6 double-blind (DBPCFC) food ingestion challenges with milk; ^○^: positive skin response to milk extract.

**Table 3:** Agreement between OFICH and other diagnostic parameters.

	History + Skin + (*n* = 32)	History − Skin + (*n* = 5)	History + Skin − (*n* = 17)	Total (*n* = 54)
OFICH (*n* = 54)				
(i) 39 positive responses	26 (48%)	2 (4%)	11 (20%)	39 (72%)
(ii) 15 negative responses	6 (11%)	3 (6%)	6 (11%)	15 (28%)

Total	32 (59%)	5 (10%)	17 (31%)	54 (100%)

**Table 4:** Survey of detailed agreement between asthmatic response types to milk ingestion challenge (OFICH) recorded in patients of both the groups and other diagnostic parameters (disease history and skin tests) for milk.

	Asthmatic responses to OFICH with milk		
	Total	LAR	NAR
Patients	*n* = 54	*n* = 39	*n* = 15
Group I. (*n* = 46)			
(i) History + Skin +	26	21	5
(ii) History + Skin −	15	9	6
(iii) History − Skin +	5	2	3

	46	32	14

Group II. (*n* = 8)			
(i) History + Skin +	6	5	1
(ii) History + Skin −	2	2	0
(iii) History − Skin +	0	0	0

	8	7	1

Group I: patients with already existing bronchial asthma to inhalant allergens and additional suspicion of milk allergy.

Group II: patients in whom the milk allergy has been suspected to be a sole cause of their bronchial complaints.

+: suspect or positive; −: unknown or negative; OFICH: open food ingestion challenge with milk.

**Table 5:** Survey of the asthmatic responses to inhalant allergens in patients developing positive and negative asthmatic response to OFICH with milk.

	Patients developing asthmatic response to OFICH with milk		
	Total	LAR	NAR
	*n* = 54	*n* = 39	*n* = 15
Group I (*n* = 46)			
Total bronchial challenge with inhalant allergens	**72**	**53**	**19**
(i) Positive asthmatic response	52	35	17
(ii) Negative asthmatic response	20	18	2
Group II (*n* = 8)			
Total bronchial challenges with inhalant allergens	**23**	**20**	**3**
(i) Positive asthmatic response	3	1	2
(ii) Negative asthmatic response	20	19	1

Total BPT with inhalant allergens	95	73	22

Group I: patients with already existing bronchial asthma to inhalant allergens and additional suspicion of milk allergy.

Group II: patients in whom the milk allergy has been suspected to be a sole cause of their bronchial complaints.

+: positive; −: negative; OFICH: open food ingestion challenge with milk.

**Table 6:** Correlation between OFICH and DBPCFC.

	DBPCFC (*n* − 54)		
	Positive (*n* = 40)	Negative (*n* = 14)	Total
OFICH (*n* = 54)			
(i) 39 positive OFICH	38**	1	39
(ii) 15 negative OFICH	2	13*	15

Total	40	14	54

OFICH: open food ingestion challenge; DBPCFC: double-blind placebo controlledfood challenge; Statistical significance: * < 0.05; ** < 0.01.
